# Feasibility of a Secure Wireless Sensing Smartwatch Application for the Self-Management of Pediatric Asthma

**DOI:** 10.3390/s17081780

**Published:** 2017-08-03

**Authors:** Anahita Hosseini, Chris M. Buonocore, Sepideh Hashemzadeh, Hannaneh Hojaiji, Haik Kalantarian, Costas Sideris, Alex A.T. Bui, Christine E. King, Majid Sarrafzadeh

**Affiliations:** 1 Department of Computer Science, University of California Los Angeles, 4732 Boelter Hall, Los Angeles, CA 90095, USA; anahosseini@cs.ucla.edu (A.H.); cbuonocore@ucla.edu (C.M.B.); sepidehhz@cs.ucla.edu (S.H.); kalantarian@cs.ucla.edu (H.K.); sidcostas2000@gmail.com (C.S.); majid@cs.ucla.edu (M.S.); 2 Department of Electrical Engineering, University of California Los Angeles, 56-125B Engineering IV Building, 420 Westwood Plaza, Los Angeles, CA 90095, USA; hannahojaiji@ucla.edu; 3 Department of Radiological Sciences, University of California Los Angeles, 924 Westwood Blvd., Suite 420, Los Angeles, CA 90024, USA; buia@mii.ucla.edu

**Keywords:** wireless health systems, mobile health, smartwatch, pediatric asthma, HIPAA compliant security

## Abstract

To address the need for asthma self-management in pediatrics, the authors present the feasibility of a mobile health (mHealth) platform built on their prior work in an asthmatic adult and child. Real-time asthma attack risk was assessed through physiological and environmental sensors. Data were sent to a cloud via a smartwatch application (app) using Health Insurance Portability and Accountability Act (HIPAA)-compliant cryptography and combined with online source data. A risk level (high, medium or low) was determined using a random forest classifier and then sent to the app to be visualized as animated dragon graphics for easy interpretation by children. The feasibility of the system was first tested on an adult with moderate asthma, then usability was examined on a child with mild asthma over several weeks. It was found during feasibility testing that the system is able to assess asthma risk with 80.10 ± 14.13% accuracy. During usability testing, it was able to continuously collect sensor data, and the child was able to wear, easily understand and enjoy the use of the system. If tested in more individuals, this system may lead to an effective self-management program that can reduce hospitalization in those who suffer from asthma.

## 1. Introduction

Pediatric asthma affects nearly six million children in the United States [1] and significantly impacts their quality of life and healthcare-related costs. Recent statistics from the National Health Interview Study have found that 9% of U.S. children currently suffer from asthma [2], and it is the leading cause of hospitalization for those under the age of 15. Specifically, nearly 10% of children diagnosed with asthma go to an emergency room each year [3,4], and it is the leading cause of student absenteeism, causing upwards of 14 million school days lost per year [5,6]. Pediatric asthma is also a very costly disease, as it is estimated that healthcare costs for a child with asthma average $1039 per year [7,8].

It is clear that there is a significant need to alleviate symptoms and continuously manage this disease. Mobile Health (mHealth) systems provide a potential platform for continuous symptom management through patient education, medication adherence and increased monitoring accuracy. While most previous asthma mHealth applications (apps) have focused on these aspects of mobile health [9], much of the information captured is typically through e-diaries and self-reports rather than automated sensor-based data collection. A recent review [10] demonstrated that the reliance on e-diaries and an absence of passive sensing (i.e., sensors that do not require people to intervene) was associated with reduced compliance and adherence. These observations suggest that there is a need for better integration of wireless sensing and communication protocols with asthma apps to reduce the amount of e-diary requests from the patient. If successful, a passive wireless sensing mHealth system may improve asthma symptom monitoring and awareness of asthma attack risks through individualized real-time recommendations within the community.

### 1.1. Related Work

With the advancement of low-cost wireless sensors, as well as smart phones and smart watches, community-based monitoring and interventions through mHealth systems have become increasingly adopted by healthcare professionals and the general public [11]. For those who suffer from asthma, mHealth systems can help patients improve their symptoms through patient education and monitoring [12,13,14,15,16]. Prior mHealth systems for the treatment of asthma have focused on monitoring of asthma through wireless sensing [14,17], self-reports [15,16] and online sources [14,15,16,17]. These previous studies have found a positive impact on patient education and adherence. However, in order to detect asthma symptoms in real time, mHealth systems require continuous remote sensing of the patient’s physiological state, their environment, cognitive health, physical activity levels, as well as their medication use through the use of all of these types of data collection.

As described in Anantharam et al. [14,17], online Internet sources were combined with wireless sensing to provide continuous monitoring of a variety of potential asthma exacerbations. Specifically, sources for environmental information including atmospheric, traffic and pollutant metrics were collected from WeatherForYou.com, Pollen.com and AirNow.gov to obtain real-time information about the current weather, air pollution and pollen levels based on the individual’s location determined through GPS on the mobile phone. These data were then synchronized with wireless environmental and activity sensors to provide an enriched set of information while minimizing user involvement. In addition, self-reports through scheduled ecological momentary assessments, such as those presented in the Asthma Mobile Health study [15,16], were used to assess asthma symptoms in combination with these online Internet sources. However, this app did not utilize any wireless sensing and was only available for iPhone users [16], who typically have a higher education and income level than other smart phone users [18].

The platform utilized in this study takes advantage of the online Internet sources used in both of the above studies [14,15,16,17] and those reviewed in Huckvale et al. [19]. In addition, our study focuses on utilizing wireless sensing and Android-based smart phones and smart watches to understand the risk of an asthma attack, as this provides an open-source operating system framework that is less expensive and thus more widely available to potential users with lower income and education levels. Furthermore, our system uses the enriched datasets collected through real-time wireless sensing and online Internet sources to develop a personalized model that includes a wide range of signals that can detect both physiological and environmental asthma exacerbations. Since current asthma mHealth systems such as those presented above [14,15,16,17] and reviewed in Huckvale et al. [19] do not address security concerns nor comply with Health Insurance Portability and Accountability Act (HIPAA) standards, the system presented here also maintains HIPAA-compliant security and privacy through a hybrid cryptographic framework. Finally, this platform focuses on asthma in pediatric patients, rather than adults, the target population of other previous research on mHealth systems for the self-management of asthma [14,15,16,17,19], which is a much more vulnerable population that could greatly benefit from mHealth asthma systems.

Children with asthma often require individualized continual treatment and symptom control. This is rather complicated, however, as symptoms and exacerbations can vary during the day (e.g., daytime and nighttime symptoms, activity levels) and throughout the year (e.g., with seasons or specific allergens) [20]. The severity of a child’s asthma may also change during their life, as some children may see changes in their symptoms during adolescence (ages 10–19 years). In addition, children who are 5–12 years of age should be directly involved as much as possible during the course of their treatment and should establish goals for therapy through written asthma action plans [20]. These continually-changing plans allow the child to directly set goals for his or her treatment and thus increase their personal responsibility and problem-solving skills. Thus, treatment action plans require continuous changes, and since symptoms can vary over time, individualized and precise real-time measurements are necessary for asthma apps to provide better recommendations. The asthma app system described below provides these individualized recommendations through risk levels determined by real-time wireless sensors and analytics models developed from each individual’s physiological and environmental data.

### 1.2. Objectives

In this study, we expand upon our previous work described in Hosseini et al. [21] to determine the overall risk of an asthma attack. As mentioned in our prior work, using wireless sensors, data are collected in real time via a smartwatch app. The data on the smartwatch are then sent via HIPAA-compliant encryption to a HIPAA compliant cloud for real-time integration with online sources collected via Representational State Transfer Application Programming Interfaces (RESTful APIs). After performing a combination machine learning approaches, the resulting asthma risk is classified and provided to the user via child-friendly animated graphics on the smartwatch. As described below, the platform now uses improved child-friendly dragon animations to display the asthma risk level on the smartwatch to users, as well as providing a new graphical user interface on the phone for the caregiver or user to review the environmental and physiological data throughout the day. These new features were included to help children and caregivers better understand the consequences of increased asthma risk levels and symptoms and the physiological and environmental context behind their resulting asthma risk levels.

The main research objective of this study was to assess the initial feasibility and usability of the improved platform in individuals who suffer from asthma. To determine whether the system was valid under various asthma exacerbation conditions that cause asthma symptoms such as exposure to smoke, high intensity physical exercise and hyperventilation, the system was tested in an adult with asthma over the course of a week. This helped assess whether it is feasible for the system to detect an individual’s current overall risk of having an asthma attack (i.e., asthma attack risk level categorized as high, medium or low) under real-world conditions in real time. Since it is not ethically responsible to expose a child to conditions that cause asthma exacerbations, usability rather than validation of the model was assessed by testing the system on a child in the community over the course of several weeks. In addition, self-reports of asthma symptoms by the child would be less reliable, and the child may or may not be exposed to any asthma exacerbation conditions during the test period. This testing was thus designed to assess whether a child can wear and use all of the sensors and smartwatch to collect continuous data and understand the feedback provided on the smartwatch app, which was assessed through an informal interview.

## 2. Materials and Methods

As described in Hosseini et al. [21], our mHealth asthma system relies on physiological and environmental wireless sensing, as well as online sources to continuously estimate the risk of an asthma attack in real time. In addition, we implemented HIPAA-compliant cryptography framework to ensure safety and privacy during use of the system. To assess the feasibility of the system’s ability to assess asthma attack risk under real-world conditions within the community, it was first tested on an adult individual with moderate asthma (female, Caucasian, middle-low socioeconomic status, age 29) under controlled asthma exacerbation conditions outside of the laboratory in her home, followed by feasibility testing in a child with mild asthma in her community and home (female, African-American, middle-high socioeconomic status, age seven). During the feasibility testing with the child, the system was deployed in the child’s home environment for a week in uncontrolled real-world conditions. Both individuals provided informed consent under the University of California Los Angeles (UCLA) Institutional Review Board (IRB 15-001402). The child’s caregiver, as well as the child provided informed consent by signing the UCLA Parent Permission for Minor to Participate in Research form and the UCLA Child (Ages 7–12) Assent to Participate in Research form, respectively.

### 2.1. System Overview

**Figure 1:**
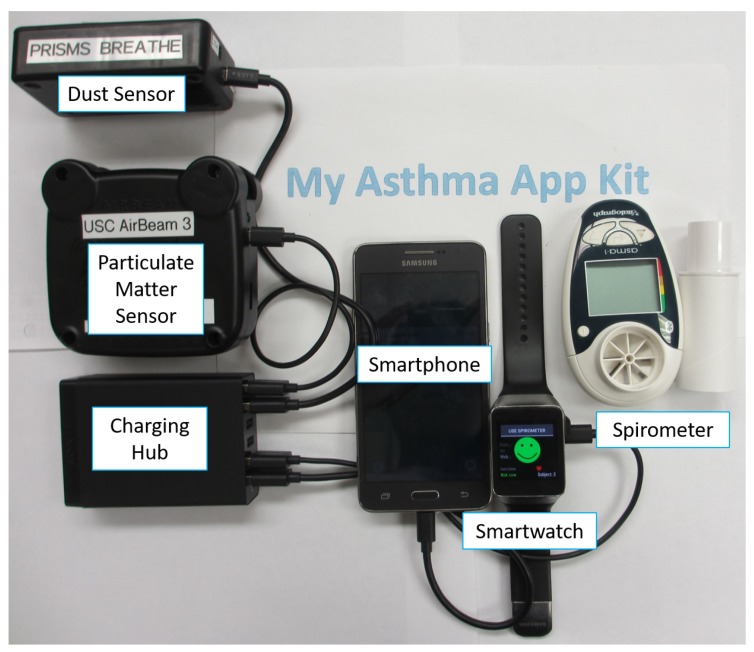
My Asthma App Kit given to individuals for real-time asthma risk assessments.

The mHealth asthma system consisted of a wireless custom-built dust sensor, a commercially-available particulate matter sensor (AirBeam, AirCasting, Habitat Map, Inc., Brooklyn, NY, USA) and a commercially-available spirometer (Asthma-1 Bluetooth Monitor, Vitalograph, Buckingham, UK; see Figure 1). These sensors sent encrypted data in real time to an Android smartwatch (Samsung Gear Live, Samsung, Seoul, Korea) via Bluetooth 4.0 Low Energy (BLE) communication. The smartwatch then combined these data with the heart rate, acceleration, gyroscope, time and location information, encrypted the combined data and sent them to the cloud. The cloud then decrypted the data and pulled online Internet sources at the nearest location and corresponding timestamp via RESTful APIs. These data were then used as input features in a real-time analytics model to estimate the overall asthma risk. The resulting risk level was then encrypted and sent back to the smartwatch in real time and displayed as an animated dragon interface. The system setup allowed the app to run for 13 h out of the maximum 16 h of available smartwatch use (the average battery life of the watch if the app was not being used). In addition to the smartwatch user interface, data trend lines were made available on a smartphone (Samsung Grand Prime, Samsung, Seoul, Korea) for the user or caregiver to be able to review the incoming data.

### 2.2. Wireless Sensors

The wireless sensors collected data using a closed HIPAA-compliant cryptography design. To this end, the data were encrypted on the firmware using 128-bit Advanced Encryption Standard (AES) cryptography and sent to the smartwatch using the BLE stack. This level of encryption was deemed sufficient, as BLE encrypts data using 128-bit AES [22], which meets the HIPAA encryption requirement level to adequately protect electronic protected health information [23].

#### 2.2.1. Environmental Sensors

In order to collect local environmental changes that can affect asthma symptoms, two wireless sensors were integrated with the asthma app: a custom-built wireless dust sensor and a commercially-available particulate matter sensor (AirBeam). Both sensors were wearable and <10 cm in size and can be worn around the belt or carried in a child’s pocket or back pack with the sensor intake facing the surrounding environment (see Figure 1). In addition, the custom-built dust sensor measures dust density measurements, while the particulate matter sensor collected fine particulate matter data of small air pollutant particles that are smaller than 2.5 μm in diameter (PM 2.5). Both sensors also were able to collect local temperature and humidity data from built-in sensors.

**Figure 2:**
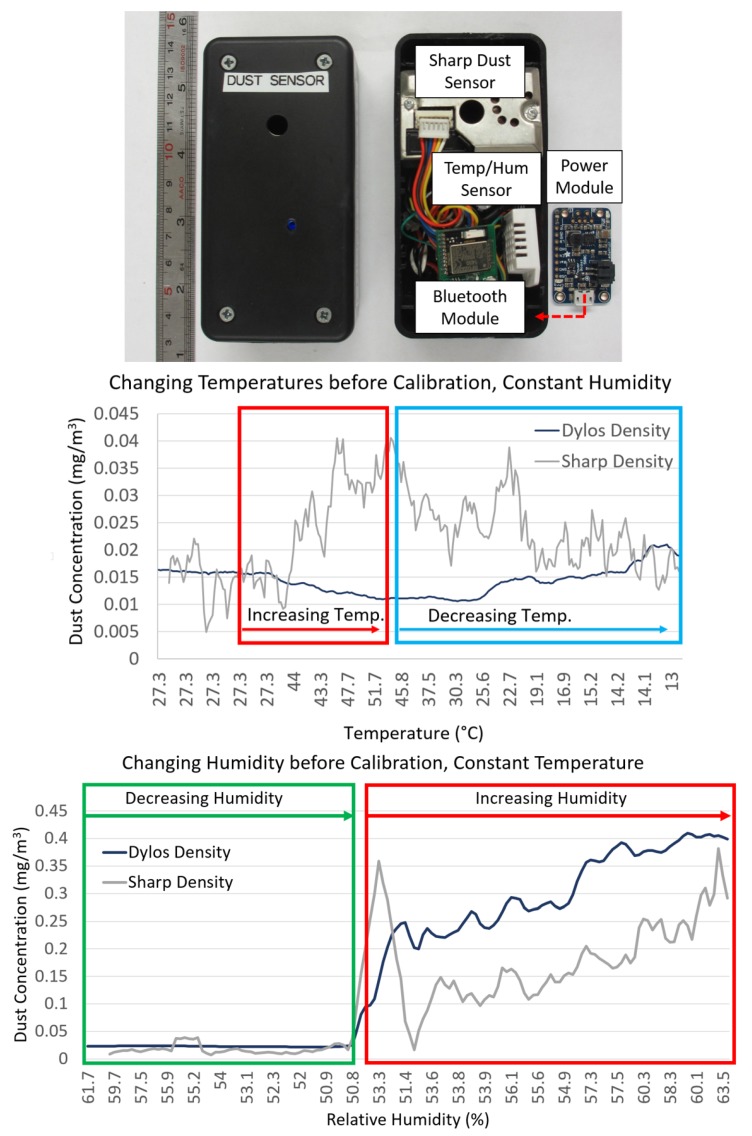
(**Top**) Custom-built wireless dust sensor for dust density data prototype design and circuit diagram. Calibration experiments performed: (**Middle**) Dylos and dust sensor exposed to increasing and decreasing temperatures while humidity and dust concentrations are kept constant; (**Bottom**) Dylos and dust sensor exposed to increasing humidity while temperature and dust concentrations are kept constant.

The custom-built wireless dust sensor for dust density measurements was developed in our laboratory using a compact optical dust sensor (Sharp Corporation, Osaka, Japan; see Figure 2) and was validated against a Dylos professional air quality monitor (DC1100 Pro, Dylos Corporation, Riverside, CA, USA) for sensitivity and accuracy under several real-world air quality conditions. For the dust sensor to be portable, it was integrated with a BLE module (ARM Cortex-M0 microcontroller with integrated Bluetooth 4.0 LE, RFDigital Corporation, Hermosa Beach, CA, USA), power supply board (PowerBoost 500C, Adafruit Industries, New York, NY, USA) and a temperature and humidity sensor (DHT22, Adafruit Industries, New York, NY, USA). It was then calibrated against the Dylos air quality monitor under varying temperatures and humidities so that the measured dust densities are accurate under varying conditions (see Figure 2). The calibration experiments resulted in the following equation and coefficients to mitigate the effects of temperature and humidity changes on dust density:

(1) $$\rho_{c}=\left\{\begin{array}{c} 5.4\times 10^{-4}x-0.15;\Delta H=0,\Delta T=0\\ \alpha_{H}H+\beta_{H}-\left(\alpha_{T1}T+\beta_{T1}\right);\Delta H>0,\Delta T>0\\ \alpha_{H}H+\beta_{H}-\left(\alpha_{T2}T+\beta_{T2}\right);\Delta H>0,\Delta T<0\\ 5.4\times 10^{-4}x-0.15-\left(\alpha_{T1}+\beta_{T1}\right);\Delta H<0,\Delta T>0\\ 5.4\times 10^{-4}x-0.15-\left(\alpha_{T2}+\beta_{T2}\right);\Delta H<0,\Delta T<0 \end{array}\right.$$

where $\rho_{c}$ is the dust density concentration, *x* is the raw voltage value from the dust sensor, $\alpha_{H}$ is $2.8\times 10^{-3}$, $\alpha_{T1}$ is 0.0005, $\alpha_{T2}$ is 0.0002, $\beta_{H}$ is 0.1647, $\beta_{T1}$ is 0.0133 and $\beta_{T2}$ is 0.0329.

The wireless sensor environmental data collected from the mHealth asthma system are particularly important in asthma research as small particles can be inhaled deeply into the lungs, causing increased constriction and inflammation, which can lead to chronic phlegm, chronic cough and bronchitis [24]. Dust density, which measures the density of particles of varying sizes including those greater than 10 μm in diameter, is able to capture larger particles related to indoor allergens, such as those due to dust mites, cockroaches or pets, which can exacerbate asthma symptoms in children [25]. Note that these particles are much larger than those that would be present on clothing during the use of the sensor, so only these larger particles due to potential environmental exacerbations are detected by the sensor. Finally, temperature and humidity affect asthma symptoms, as increased temperature and humidity levels can irritate the airways and lead to inflammation [26].

#### 2.2.2. Spirometer

The Vitalograph Asthma-1 electronic peak flow meter (Vitalograph Ltd., Lenexa, KS, USA) was used to obtain spirometry data from the users in real time. Spirometry is the most important physiological measurement for the treatment of asthma, as it determines the severity of the individual’s asthma [20]. Furthermore, the wireless spirometer was used over examining lung function via a microphone on the smartphone [27] or smartwatch, as this sensor is clinically validated and FDA-approved. To measure spirometry in the community, the PEF and FEV1 were obtained. These values are crucial for the clinical assessment of asthma because a reduction in PEF and FEV1 compared to age and height-matched healthy individuals’ averages elucidates the overall severity of the asthma at a particular time [20].

Immediately after an individual performed spirometry using the Vitalograph sensor, the PEF and FEV1 data were sent to the cloud. To this end, the sensor was connected to the smartwatch by pressing the “Use Spirometer” button on the watch, which then listened for incoming data and uploaded the information using the information provided in the device’s API developer’s toolkit. The data were then combined with any other incoming sensor data from the environmental sensors and smartwatch and then sent to the cloud for real-time analytics.

### 2.3. Asthma App

The Samsung Gear Live Android smartwatch was used to provide real-time data collection and feedback to the individual. The watch was linked with a smartphone (Samsung Grand Prime) to provide real-time data trend lines to the caregiver and users. A smartwatch was used with the phone because children with asthma are less likely to carry a phone during play and exercise [28], known asthma exacerbations.

**Figure 3:**
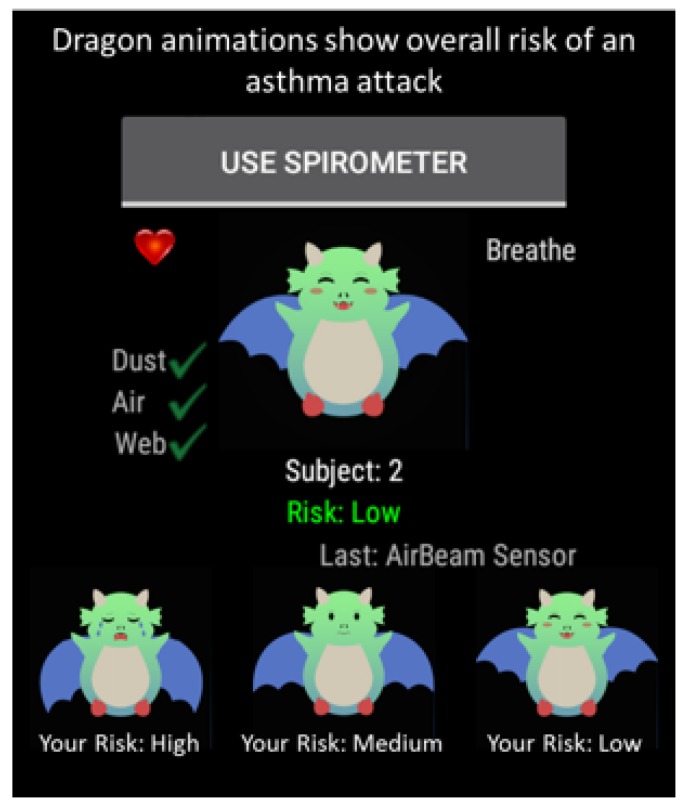
Asthma app user interface on the smartwatch. The dragons are animated, showing a happy bouncing dragon at a low asthma risk, a neutral face and slower bouncing dragon at a medium risk and a crying slow moving dragon at a high asthma attack risk level.

The wireless sensors sent dust density, PM 2.5, and spirometer data to the smartwatch via encrypted BLE communication. This data were then appended to acceleration, gyroscope, heart rate, time and location data pulled from the smartwatch (Figure 1). The smartwatch encrypted and transmitted the combined data to the cloud through WiFi or 4G LTE communication. The cloud then decrypted the data, which was then inputted into the machine learning analytics model to calculate the asthma attack risk. The resulting risk level was then sent with the same secure method from the cloud to the smartwatch and displayed (Figure 3) as a happy (low risk), neutral (medium risk) or crying (high risk) animated dragon graphic.

The animated dragon graphics were designed specifically for children, as they are more engaging and can be easily interpreted as different risk levels associated with the risk of an asthma attack. It has been found in previous research that video games and animations provide a channel for health behavior change experiences in an engaging and entertaining format [29,30] that can positively affect a child’s health outcome. Thus, we decided to use an animated dragon that cries and does not bounce very high when a child is at a high risk of an asthma attack, bounces with a neutral face when the child is at a medium risk and bounces very high with a large smile when the child is at a low risk of an asthma attack. By associating these animations with asthma risk levels, we hope to engage the child such that his/she is educated through the dragons on how his/her current physiological and environmental asthma triggers can have consequences such as worsened asthma symptoms that can potentially lead to an asthma attack.

### 2.4. Smartphone App

**Figure 4:**
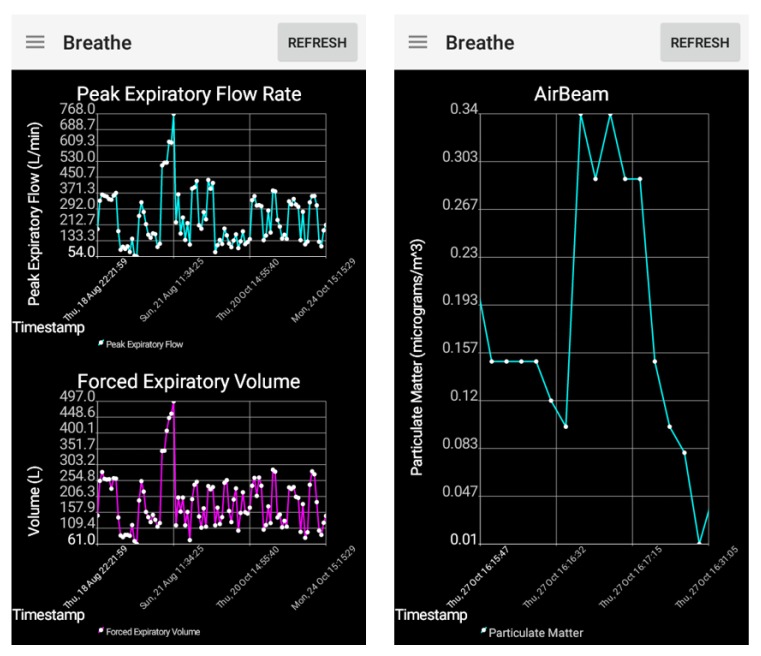
Data trend lines for the spirometer and AirBeam sensors displayed in real time on the smart phone. (**Left**) PEF and FEV1 measurements taken by the spirometer, (**Right**) PM 2.5 measurements taken by the AirBeam.

To allow the caregivers and users to visualize their environmental and physiological data in relationship to their asthma symptoms, graphical interfaces for the wireless sensor data were developed on the smartphone (Figure 4). These data trend lines allow users to visualize the data and output risk levels in real time for each sensor type, as well as review their data over the last several hours. The user is able to scroll and zoom onto points of interest for each sensor. If the user wanted to keep certain trend lines to review throughout the day, they were able to do so, or could update their trend lines by refreshing the graphical interfaces. This allowed the caregiver or user to better understand which type of asthma trigger (e.g., environmental due to dusty conditions, physiological due to a high heart rate) was causing an increase in the asthma symptoms currently or at specific instances throughout the day.

The smartphone interface also allows physicians and study coordinators to register the asthma app to specific users. This was accomplished by having the app first display a screen that required the physician to enter a valid clinician email and de-identified subject ID when the app is first downloaded on the smartphone and smartwatch. The interface then opens the home screen where data trend line pages can be opened if a valid ID and email pair are determined by the cloud. These IDs and emails were also managed by the cloud and followed HIPAA-compliant authorization, authenticity and de-identification of protected health information.

### 2.5. HIPAA Compliant Cryptography

For HIPAA-compliant data transfer, the data were encrypted using 128-bit AES. To protect the key used to decrypt the AES encryption, the Rivest-Shamir-Adleman (RSA) encryption algorithm was used to send the AES symmetric key from the smartwatch to the cloud [21]. This method uses an asymmetric (public) key to encrypt the symmetric (private) AES key. Specifically, a pair of public and private keys on both the smartwatch and cloud is generated. While the public key is known by both parties, the private key is kept secret. The smartwatch generates a private key, then uses the cloud’s generated public key to encrypt the private key and sends it to the cloud for data decryption, where it is stored in an encrypted database.

The above cryptography framework was used as Silva et al. [31] found that this technique achieves comparable performance levels to data transmission without encryption. It also offers an increase in privacy, confidentiality, integrity and authenticity of the protected health information required for HIPAA compliance [31]. Furthermore, the AES-RSA hybrid cryptography framework allows for faster encryption time in larger data transfers compared to other encryption algorithms. This is important in our application given the large amount of data collected from wireless sensors and the smartwatch.

To protect the integrity of the data, access controls via unique usernames and strong passwords requirements were implemented. In addition, audit controls were created by requiring those who register to be authorized by the administrator, and the website automatically logged the authorized user off after 10 min of unattended use to safeguard those who have access to the data [32].

### 2.6. The BREATHE Cloud Platform

The Biomedical Real-Time Health Evaluation (BREATHE) cloud platform (https://www.breatheplatform.com) used the Amazon Elastic Computer Cloud (Amazon EC2, Amazon Web Services, Seattle, WA, USA) to store and analyze the data from the asthma mHealth system. The cloud pulled the encrypted data sent from the smartphone (which was synced with the smartwatch) and decrypted the data using the hybrid cryptography method presented in the previous section. It also pulled connection and battery information to allow for real-time system debugging and reminders for sensor charging to the users. Finally, the pulled timestamp and GPS data were used to combine these data with online sources via RESTful APIs, and all data were stored at rest using 256-bit AES cypher block chaining encryption.

The online data collected via RESTful APIs described in our prior work [21] consisted of atmospheric, traffic and pollutant intensity information. Data were gathered from three online sources: AirNow.gov, Forecast.io and MapQuest.com, which used geographical coordinates and timestamps in an HTTP request to return the requested data over a secure socket layer.

### 2.7. Real Time Analytics

#### 2.7.1. Data Pre-Processing

After data transmission and decryption, which had negligible lag compared to the varying sample rates of the data, initial data manipulation was performed. First, since the gathered data contained some missing or out-of-bound values (for those sensors that provided a data point completely out of the sensor’s range to represent a missing value), samples were removed from the dataset that were considered negligible compared to the rest of the dataset. Next, in order for the accelerometer data to determine the individual’s level of physical activity, the algorithm described in Yamada et al. [33] was used. This was chosen over the widely-accepted doubly-labeled water method for estimating total energy expenditure from accelerometers, as it has a higher correlation with measured metabolic equivalents. To implement this algorithm, the vector norm of the composite acceleration ($K_{m}$) was calculated as:

(2) $$K_{m}=\sqrt{\left(1/(n-1)[Q-1/n(P)]\right)}$$

where:

(3) $$Q=\sum_{i=0}^{n}x_{i}^{2}+\sum_{i=0}^{n}y_{i}^{2}+\sum_{i=0}^{n}z_{i}^{2}$$

and:

(4) $$P={\left(\sum_{i=0}^{n}x_{i}\right)}^{2}+{\left(\sum_{i=0}^{n}y_{i}\right)}^{2}+{\left(\sum_{i=0}^{n}z_{i}\right)}^{2}$$

The variables *x*, *y* and *z* are the raw accelerometer values measured over a 5-s time window.

The dust sensor and AirBeam data were also preprocessed prior to inputting them into the analytics model such that the dust density and particulate matter were used rather than the raw voltage values. This was done by removing dropped packets, increasing the sensitivity by scaling when the data deviate from the baseline values and using a moving window of 6 s to smooth the data. Finally, for the spirometer sensor, the percent from baseline value was calculated after conducting three baseline measurements while at rest and experiencing no asthma symptoms prior to use of the app in the community. This was done rather than using raw values as an input feature, as the American Lung Association uses this method to develop asthma management plans [34]. For the heart rate data, the heart rate reserve was calculated using the following equation:

(5) $$HRR=HR_{max}-HR_{rest}$$

where $HR_{max}=208-0.7\left(\mathrm{age}\right)$ [35], the maximum heart rate that closely predicts this value in children, and $HR_{rest}$ is the resting heart rate taken during baseline assessment.

#### 2.7.2. Analytics Model

The processed data were fed into a training model to perform real-time machine learning. To this end, a random forest classifier was used to classify data into three different high, medium and low risk levels. This type of classifier was chosen as it uses bootstrapping aggregating to improve the predictive accuracy and controls for over-fitting. Furthermore, this method had the best cross-validation performance when compared to other machine learning methods such as naive Bayes. It has also been employed and found to be highly accurate in several previous mHealth- and healthcare-related studies [36,37,38,39].

A training model was first generated offline given the ground truth labels of the Asthma Control Test (ACT) scores every hour rather than the last four weeks so that a sufficient number of labels could be collected (http://www.asthmacontroltest.com/; see the next sections for further details). Furthermore, the dust, activity and heart rate data were continuously collected, and baseline values were collected when the subject was at rest in a low pollution environment. This was then used to perform real-time predictions of the risk level. Then, during real-time classification, a fixed size window of the last reported data was compared to the training model. For the dust and AirBeam sensor data, this window was used to avoid sudden changes in dust readings calculated from Equation (1); for the acceleration and heart rate data, it was used to compute the energy expenditure using Equations (2)–(4) and heart rate reserve using Equation (5), respectively. The resulting features to assess the risk level were: percent FEV1, percent PEF, dust density, particulate matter, temperature, humidity, heart rate reserve, total energy expenditure, ozone, pressure, cloud cover, wind speed, precipitation probability, precipitation intensity, traffic density and Air Quality Index (AQI).

#### 2.7.3. Validation of Asthma Risk

To test the feasibility of the current system under controlled conditions, the adult with asthma collected data using the asthma mHealth system over the course of five days while exposed to a wide range of asthma exacerbations in real-world conditions such as running outside and smoke due to cooking. The performance of the risk model was assessed by having the adult perform the complete ACT every hour, and spirometry was performed every minute for 20 min so as to collect as many data points as possible while preventing hyperventilation due to excessive exhalations. This self-report was used as the ground truth label, because it is a widely-used clinical measure for asthma control and has been used to validate previous asthma apps [19]. Furthermore, it is available for our target population of children 4–11 years old. Note, however, that the child was not asked to perform spirometry every hour, as we were more concerned with usability while in the community. Instead, the child was asked to perform spirometry measurements at baseline to generate the individualized risk model, then whenever she felt any symptoms or desired to use the spirometer so as to not burden her with excessive spirometry measurements.

#### 2.7.4. Evaluation Measures

To evaluate the modeling results given the ACT data, we plotted the ground truth ACT scores against the predicted value for a portion of the test samples. To this end, these scores were separated into high, medium and low risk level ground truth labels given that if a score is 19 or less, then the asthma symptoms may not be as well controlled as they could be, and if the score is 15 or less, then asthma is poorly controlled, as stated in the ACT document. Thus, scores from 25–19 were considered as a low risk level, 15–19 as a medium risk level and less than 15 as a high risk level. Furthermore, we used 10-fold cross-validation while applying grid search in all methods to obtain optimal results that fit best with the ACT results, as almost every method used in this study contained hyper-parameters that required tuning. To this end, the data and corresponding labels (ACT scores) were randomly partitioned into 10 equally-sized subsamples, and each subsample was used for validation, while the rest were used as training data. This process was repeated 10 times (or folds), and the results from the folds were combined to produce an accuracy estimation. Note that for the three-class classification presented in this study, the chance level classification of randomly predicting the correct risk level is 33%.

The usability of the system was also evaluated using an informal interview. To this end, after the child used the system within the community for several weeks, she was asked by the researchers whether there were any issues with wearing the sensors and smartwatch during her daily activities. After initial explanation that the dragon animation on the smartwatch provides her with her level of risk of an asthma attack given the environment and her behaviors, she was then asked after the usability experiment if she understood what the animations meant and how they correspond to her asthma risk level and what this meant in terms of her current health state. She was also provided with smiley face graphics (green happy face corresponding to a low risk, yellow neutral face for a medium risk and red sad face for a high risk) presented in our prior work [21] and asked which graphics she preferred and could more easily understand. Finally, she was asked whether she was able to carry all sensors around with her, wear the smartwatch throughout the day (her caregiver would charge the devices at night for her) and whether she could perform the spirometry measurements, as well as how many times a day she collected these data.

## 3. Results

### 3.1. Adult Feasibility Testing

**Figure 5:**
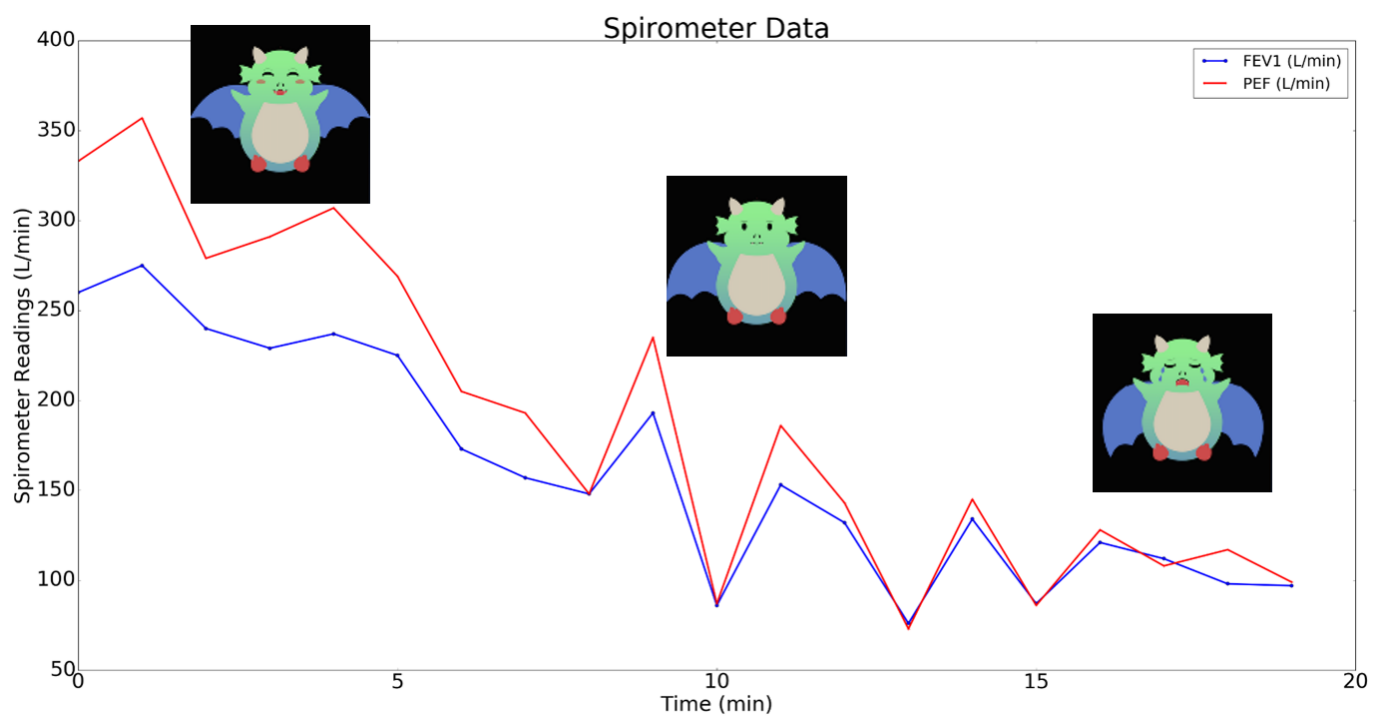
PEF and FEV1 spirometer readings and corresponding asthma risk labels when the adult individual entered a smoky room.

During the feasibility testing over the course of five days for the adult with asthma, it was found that the dust sensor, AirBeam and spirometer sensor were able to accurately send dust density, particulate matter, Peak Expiratory Flow (PEF) and Forced Expiratory Volume in one second (FEV1) readings to the cloud. For example, as seen in Figure 5, the spirometer PEF and FEV1 readings decreased when the adult was exposed to smoke. The individual with asthma also reported an average PEF value of 320.6 ± 30.3 L/min during instances of no asthma symptoms and little to no asthma risk.

The analytics model to determine the overall asthma risk level had an accuracy of 80.10 ± 14.13% N = 59 asthma risk level samples) after performing 10-fold cross-validation (chance level: 33.33%). The classifier also performed in real time, having only a 5-s lag between data collection and risk decisions displayed on the smartwatch, which was mainly due to averaging accelerometer values to reliably estimate energy expenditures. As seen in Hosseini et al. [21], FEV1, PEF, dust density and heart rate were the most important features for determining the individual’s asthma risk level under varying types of asthma exacerbations such as physical activity and exposure to smoke. Thus, the wireless sensors were most important in predicting the overall risk of an asthma attack. Furthermore, when compared to a pulse oximeter (Onyx II, Nonin Medical Inc., Plymouth, MN, USA), the heart rate data had a 96.74% accuracy under lying, sitting, standing, walking and running conditions, demonstrating that it was accurate during these feasibility tests.

### 3.2. Child Usability Testing

**Figure 6:**
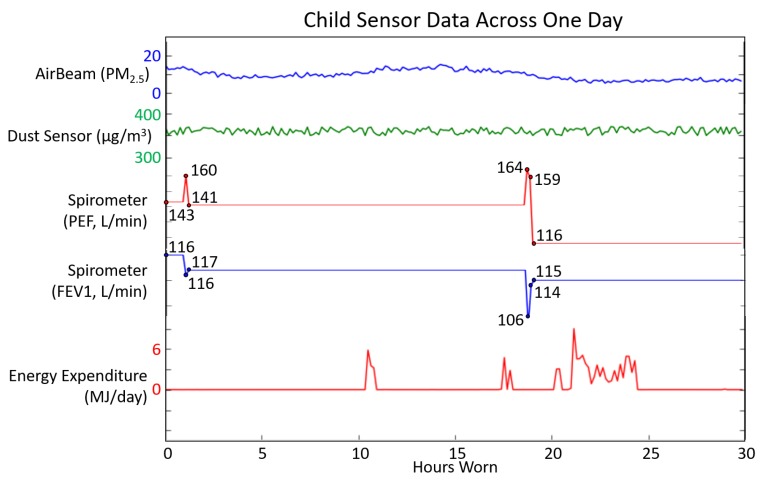
Data from select sensors during the child’s usability testing across one day of use. Spirometer values are displayed, along with sensor ranges.

The asthma app was also tested for usability by a child with mild asthma in the community for several weeks. As seen in the example of data collected in Figure 6, sensors continuously collected data in real time throughout the week and displayed an appropriate risk level of a low risk throughout the experiment. This is due to the child not experiencing any asthma symptoms during the community-based testing. Note that the child did not experience any symptoms during usability testing, and all resulting asthma risk levels determined during the several weeks of testing were classified as low risk. This corresponds to zero false positive risk changes to a higher risk level throughout the testing period; although, the validity of the system must be determined through the adult experiments, since we were unable to obtain all categories of risk level during the child testing.

The informal interview performed after usability testing was completed determined that the child was able to wear all sensors continuously, perform spirometry several times a day (typically twice a day) and could easily interpret the risk level using the animated dragon graphics. Since she did not experience any symptoms nor entered any high risk environmental conditions (e.g., walked past someone smoking, entered a smoky kitchen), she was able to understand that her animated dragon graphic never changed from a happy dragon. In addition, during the interview she was presented with both the three different dragon animations and the three faces graphics (the original smiley, neutral and sad faces presented in Hosseini et al. [21]), and she mentioned that she preferred the dragons graphics over the smiley faces to show her the current asthma attack risk level. She was also able to understand that each of these graphics corresponded to her current asthma attack risk level and what this meant to her current health state. Finally, the child mentioned that she was able to easily perform the spirometry measurements using the wireless spirometer and that she enjoyed receiving immediate feedback about what her lung function was at the time.

## 4. Discussion

The feasibility testing on the adult with asthma under different asthma exacerbation conditions demonstrated that the prediction model was able to accurately determine the risk of an asthma attack. The model had an 80% prediction accuracy across the three risk classes, and this can be improved with further samples. Furthermore, the important features to predict asthma risk were multifaceted, as both environmental and physiological features were needed to predict asthma risk levels. The heart rate reserve was a particularly interesting important feature, as it highlights the need for future asthma apps to use Photoplethysmography (PPG) sensors so that the risk can be better predicted.

During testing on the child, the system was able to continuously collect wireless sensor data over several weeks and provide enjoyable feedback she could easily understand. In the interview, the child reported no asthma symptoms during the entire test period, and the model was able to provide appropriate low asthma risk levels, thus accurately predicting the risk level throughout the entire test. When questioned about the usability, she mentioned that she enjoyed the dragon interface better than the previous smiley face image presented in Hosseini et al. [21], and she was able to easily interpret the risk level. This highlights the importance of easy to understand and enjoyable graphics when designing self-management health programs for pediatric populations. In addition, the results of the interview determined that continuous use and carrying of the sensors did not burden the child, as she was able to use the spirometer appropriately when she desired (typically twice a day) and was able to wear the smartwatch and environmental sensors during the entire period, allowing for continuous real-time streaming of physiological and environmental data.

These two studies on the child and adult were able to assess the feasibility of the prediction model to assess asthma attack risk level, as well as the usability of the system to collect physiological and environmental wireless sensor data from a child in the community. The studies have provided us with a better understanding of which features are most important in designing a prediction model for asthma attack risk and which sensors are required to monitor individuals with asthma in the community. For example, heart rate PPG sensors should be included in future asthma mHealth systems, as this is an important feature for classification. Furthermore, the wearability of the sensors and their ability to continuously collect data in the child suggest that this system design may be successful in predicting asthma attack risk level in children for a wide range of asthma attack exacerbation types, such as exposure to smoke and physical exercise. Thus, future research is necessary on this system to assess its usefulness and feasibility of determining asthma attack risk in a larger population of children who suffer from asthma. Details on how this future research will be conducted are further described below.

A limitation of this study is that the system was only tested on an adult and a child individual. The feasibility of assessing asthma attack risk must be further tested in children rather than an adult individual, although for ethical reasons, formal testing under controlled settings that cause asthma exacerbations in children may be difficult. Furthermore, the usability of the system requires further testing among more children with asthma to determine if children can wear the wireless sensors and understand the smartwatch user interface to assess their asthma attack risk level and what this means to their health. However, the promising results from these two experiments suggest that this asthma mHealth system has ideal components required to predict asthma risk under varying real-world conditions while improving compliance during long-term use, justifying formal validity and usability testing in a larger pediatric cohort.

Future research will include testing the system on more children who suffer from asthma for feasibility and usability and to improve the generalizability of the risk model by including more training samples from these tests. These experiments will be conducted during a formal clinical trial funded by the National Institute of Biomedical Imaging and Bioengineering (NIBIB) Los Angeles Pediatric Research using Integrated Sensor Monitoring Systems (PRISMS) Center. Future developments will also include providing action based on the asthma risk level, gamified incentives to encourage adherence and ecological momentary assessments for information that cannot be measured by passive sensing, such as perceived stress. The infrastructure developed will also be used to standardize asthma apps across platforms. This will be done through our current collaboration with Open mHealth (http://www.openmhealth.org/), a non-profit organization that focuses on the standardization, integration, storage, processing, visualization and sharing of mHealth data for use in existing electronic health applications such as Epic (Epic Systems Corporation, Verona, WI, USA).

## 5. Conclusions

The study described here presents a significant expansion of our prior work [21] to develop an end-to-end wireless platform that may inform children of their risk of an asthma attack through easy to understand animations on a smartwatch and data visualization on a smartphone. The feasibility testing in an adult with asthma determined that the system may be able to accurately determine asthma risk level under known asthma exacerbation conditions that are both environmental and physiological. The usability testing of the system in a child in the community over the course of several weeks determined that children may be able to wear smartwatches and carry or wear the environmental and physiological wireless sensors used to continuously assess asthma attack risk levels, as well as understand the child-friendly animated feedback on the smartwatch app. These encouraging results validate the need for further testing of the system in a larger pediatric cohort under a planned formal clinical trial with the NIBIB PRISMS Center. If proven feasible and usable by children who suffer from asthma during this trial, this system could lead to an effective community-based self-management care program.

## Abbreviations

The following abbreviations are used in this manuscript:

mHealth	Mobile Health
app	Application
HIPAA	Health Insurance Portability and Accountability Act
RESTful API	Representational State Transfer Application Programming Interface
PEF	Peak Expiratory Flow
FEV1	Forced Expiratory Volume in one second
PPG	Photoplethysmography
NIBIB	National Institute of Biomedical Imaging and Bioengineering
PRISMS	Pediatric Research using Integrated Sensor Monitoring Systems
BLE	Bluetooth Low Energy
AES	Advanced Encryption Standard
PM 2.5	Particulate Matter smaller than 2.5 μm in diameter
RSA	Rivest–Shamir–Adleman
BREATHE	Biomedical Real-Time Health Evaluation
ACT	Asthma Control Test
AQI	Air Quality Index
